# Three Signs to Help Detect Sjögren’s Syndrome: Incidental Findings on Magnetic Resonance Imaging and Computed Tomography

**DOI:** 10.3390/jcm12206487

**Published:** 2023-10-12

**Authors:** Yukinori Takagi, Ikuo Katayama, Sato Eida, Miho Sasaki, Toshimasa Shimizu, Shuntaro Sato, Kunio Hashimoto, Hiroki Mori, Mitsunobu Otsuru, Masahiro Umeda, Yoshihiko Kumai, Ryo Toya, Atsushi Kawakami, Misa Sumi

**Affiliations:** 1 Department of Radiology and Biomedical Informatics, Nagasaki University Graduate School of Biomedical Sciences, 1-7-1 Sakamoto, Nagasaki 852-8588, Japan; yuki@nagasaki-u.ac.jp (Y.T.); katt@nagasaki-u.ac.jp (I.K.); sato@nagasaki-u.ac.jp (S.E.); sasaki-m@nagasaki-u.ac.jp (M.S.); morih@nagasaki-u.ac.jp (H.M.); 2 Department of Immunology and Rheumatology, Nagasaki University Graduate School of Biomedical Sciences, 1-7-1 Sakamoto, Nagasaki 852-8501, Japan; t.shimizu@nagasaki-u.ac.jp (T.S.); atsushik@nagasaki-u.ac.jp (A.K.); 3 Clinical Research Center, Nagasaki University Hospital, 1-7-1 Sakamoto, Nagasaki 852-8501, Japan; shuntarosato@nagasaki-u.ac.jp; 4 Department of Pediatrics, Nagasaki University Graduate School of Biomedical Sciences, 1-7-1 Sakamoto, Nagasaki 852-8501, Japan; kunioh@nagasaki-u.ac.jp; 5 Department of Clinical Oral Oncology, Nagasaki University Graduate School of Biomedical Sciences, 1-7-1 Sakamoto, Nagasaki 852-8588, Japan; ootsuru@nagasaki-u.ac.jp (M.O.); mumeda@nagasaki-u.ac.jp (M.U.); 6 Department of Otolaryngology-Head and Neck Surgery, Nagasaki University Graduate School of Biomedical Sciences, 1-7-1 Sakamoto, Nagasaki 852-8501, Japan; ykumai426@nagasaki-u.ac.jp; 7 Department of Radiological Sciences, Nagasaki University Graduate School of Biomedical Sciences, 1-7-1 Sakamoto, Nagasaki 852-8501, Japan; toya@nagasaki-u.ac.jp

**Keywords:** Sjögren’s syndrome, salivary gland, magnetic resonance imaging, computed tomography, ranula, parotid cyst, parotid calcification

## Abstract

This study aimed to retrospectively investigate the prevalence of Sjögren’s syndrome (SS) among patients with ranulas, parotid cysts, or parotid calcifications; identify the characteristic magnetic resonance imaging (MRI) or computed tomography (CT) findings of the lesions associated with SS; and compare the SS disease stages among SS patients with the three lesion types. A total of 228 patients with the lesions were classified into SS, possible SS, and non-SS groups. The prevalence of SS among patients with ranulas, parotid cysts, or parotid calcifications was 16%, 24%, and 40%, and the rates of either SS or possible SS were 25%, 41%, and 64%, respectively. SS was associated with (i) ranulas: ≤17 mm; (ii) parotid cysts: bilateral and multiple; and (iii) parotid calcifications: in females, bilateral, multiple, parenchymal, and no coexisting calcifications in other tissues. SS patients with ranulas were significantly younger and had lower submandibular gland stage scores on MRI/CT than those with other lesions. Additionally, in 58% and 15% of SS patients with ranulas and parotid calcifications, respectively, detection of the lesions led to the diagnosis of primary SS. Therefore, recognizing the prevalence of SS among patients with these lesions and the findings associated with SS can help detect undiagnosed SS.

## 1. Introduction

Sjögren’s syndrome (SS) is a chronic autoimmune systemic disease that primarily targets the lacrimal and salivary glands and is classified as primary SS (pSS) or secondary SS depending on whether it is isolated or associated with other autoimmune diseases (e.g., rheumatoid arthritis, systemic lupus erythematosus, or systemic sclerosis). SS usually occurs in middle-aged and older women, and the main symptoms are decreased tears and salivary flow related to glandular tissue destruction. However, there are an increasing number of reports of juvenile SS in large cohorts, with a higher proportion of SS without sicca symptoms in juveniles than in adults [1,2,3,4]. A recent study reported that 2.19% of patients with pSS lack sicca symptoms and that patients with pSS without dryness are younger than those with dryness [5]. Early diagnosis of pSS and early intervention might improve prognosis; however, it is challenging to diagnose pSS in patients with few sicca symptoms. Therefore, it is important to identify signs other than xerostomia to aid in the diagnosis of pSS.

Imaging techniques such as ultrasonography (US), magnetic resonance imaging (MRI), and computed tomography (CT) allow for the visual evaluation of the salivary gland parenchyma and ducts and are less invasive than labial gland biopsy. Thus, imaging examinations are increasingly being used to aid in the diagnosis or severity assessment of SS [6,7,8,9,10,11,12,13,14], although the imaging findings have not yet been incorporated into the current classification or diagnostic criteria for SS. CT involves exposure to ionizing radiation and should be avoided frequently, whereas MRI and US are noninvasive and suitable for follow-up examination. In particular, US is well known to be simple, inexpensive, and versatile but has the disadvantage of being examiner dependent, unlike MRI and CT. The most well-known imaging findings of SS are (i) heterogeneous fatty degeneration in the parotid glands (PGs) and/or submandibular glands (SMGs) on MRI and/or CT, (ii) punctate sialectasis in PGs on MRI, and (iii) multiple hyperechoic bands and hypoechoic areas in PGs and/or SMGs on US [6,7,8,9,10,11].

Several other imaging findings have been reported to be associated with SS. Parotid cysts and parotid calcifications in SS have been documented for more than 20 years [15,16,17,18,19,20,21,22,23,24,25]. Histopathologically, parotid cysts in SS are benign lymphoepithelial lesions resulting from cyst formation by epimyoepithelial islands in lymphoepithelial sialadenitis [26]. Parotid calcifications in SS are presumably related to chronic inflammation caused by the reduced salivary flow and lymphocytic infiltration of the salivary glands, which lead to calcium deposition within the parotid parenchyma and ductal system [24]. Most recently, a ranula has been suggested as an early clinical sign of SS [27,28,29,30]. A ranula is a mucocele, defined as the extravasation of mucus within an intraoral cystic cavity, which is usually associated with the sublingual gland. The most common etiology of ranulas is trauma to the excretory duct. However, Takagi et al. reported the detection of juvenile pSS by a ranula on MRI [29]. The ranula is thought to form because of the extravasation of saliva from the damaged ductal epithelium caused by chronic inflammation in SS [28].

Ranulas, parotid cysts, and parotid calcifications are often found incidentally on CT and/or MRI examinations and may be important signs of pSS. However, to the best of our knowledge, most studies on patients with SS (SS patients) with these lesions have been case reports. Therefore, the present study aimed to investigate the prevalence of SS among patients with ranulas, parotid cysts, or parotid calcifications; to identify the characteristic imaging findings associated with SS; and to compare the SS disease stages on images of PGs and SMGs among SS patients with the three lesion types.

## 2. Materials and Methods

This retrospective study was conducted in accordance with the principles of the Declaration of Helsinki. Ethical approval was obtained from the Institutional Review Board (IRB) of Nagasaki University Hospital (#23032018). The requirement for informed consent from the study subjects was waived by the IRB due to the retrospective study design.

### 2.1. Study Population

**Figure 1:**
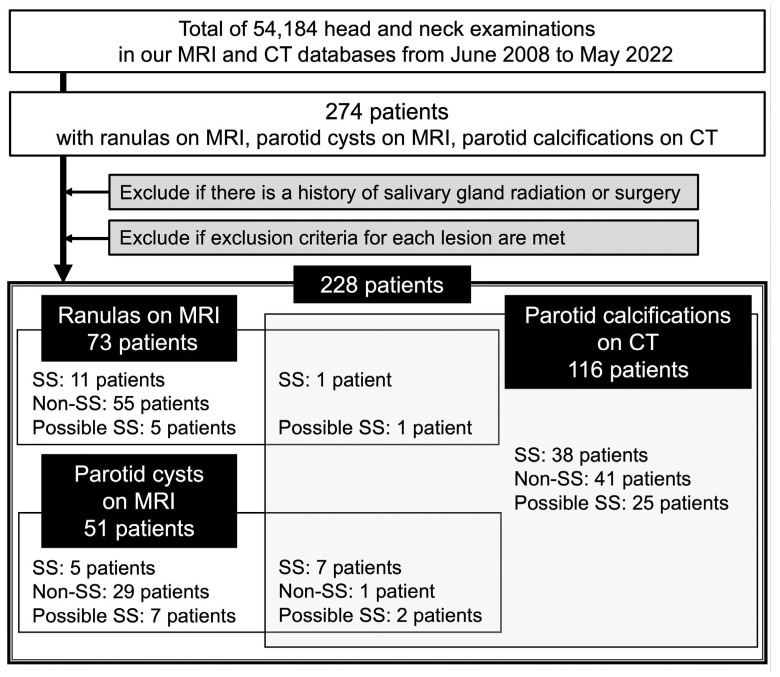
Flowchart of patient selection and classification.

To identify patients with imaging findings of ranulas, parotid cysts, or parotid calcifications, we reviewed the MRI and CT databases of individuals who underwent head and neck MRI or CT examinations at our radiology department between June 2008 and May 2022 because of suspected tumors, cysts, inflammation, vascular malformations, and other related conditions (Figure 1). The details of the inclusion and exclusion criteria for ranulas, parotid cysts, and parotid calcifications are shown in Table 1. The total study cohort comprised 240 lesions in 228 consecutive patients (168 females and 60 males; mean age, 53 years (range, 4–93 years)). Twelve patients had two lesions. Of the 240 lesions, 73, 51, and 116 met the criteria for ranulas, parotid cysts, and parotid calcifications, respectively (Figure 1). Among the 228 patients, a histologically confirmed diagnosis was made in 26 patients with ranulas, 11 with parotid cysts (all diagnosed as lymphoepithelial cysts), and 2 with parotid calcifications (both sialolithiasis).

### 2.2. MRI and CT Examinations

MRI was performed using Gyroscan Intera 1.5T Master (Philips Healthcare, Best, The Netherlands) or Skyra 3.0T (Siemens Healthineers, Erlangen, Germany). The standard protocol comprised transverse T1-weighted imaging, fat-suppressed T2-weighted imaging (fsT2WI), and diffusion-weighted imaging. Patients with insufficient fat suppression on fsT2WI were also imaged using short tau inversion recovery (STIR). Forty-six patients also underwent MR sialography of the bilateral PGs (Supplementary Table S1). Gadolinium contrast medium was administered to 21 patients.

CT examinations were performed using the HiSpeed Advantage SG CT imaging system (General Electric Medical Systems, Milwaukee, WI, USA), 64-MDCT apparatus (Aquilion 64 TSX-101A; Canon Medical Systems, Tochigi, Japan), or 320-MDCT apparatus (Aquilion ONE TSX-304A; Canon Medical Systems, Tochigi, Japan). Iodine contrast medium was used in six patients.

### 2.3. Classification of Patients into the SS, Possible SS, and Non-SS Groups

**Figure 2:**
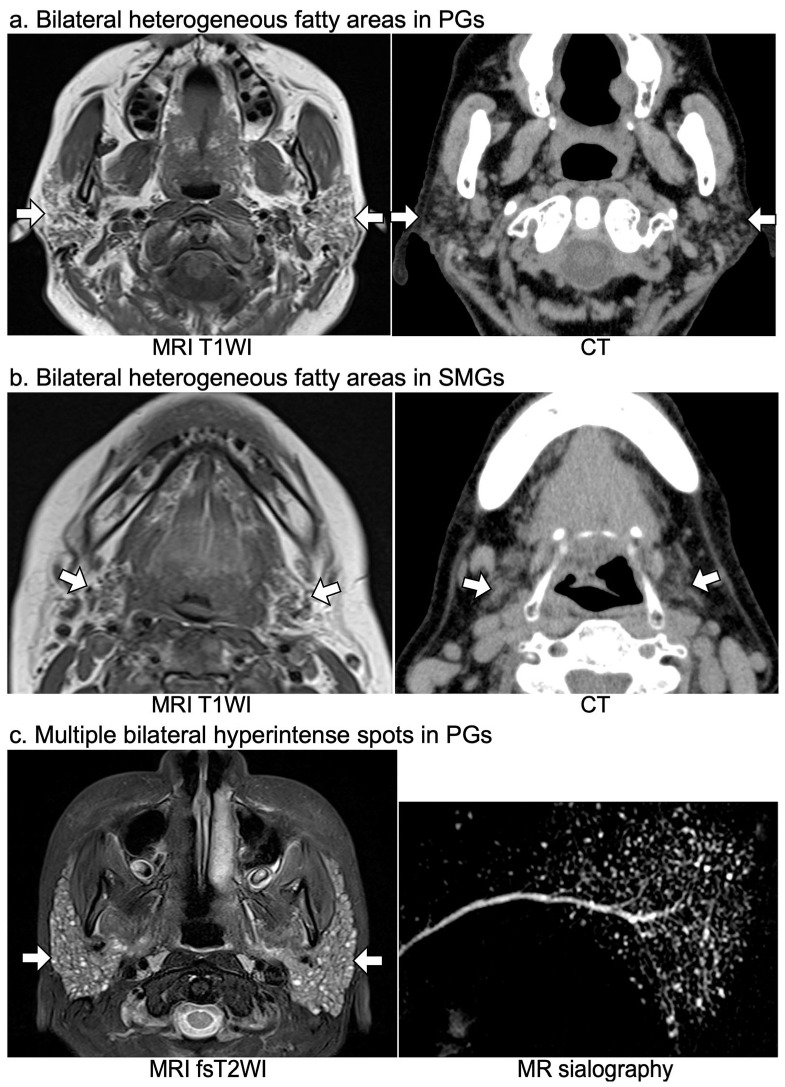
MR and CT imaging criteria for SS. The imaging criteria for SS were the presence of any of the following: (**a**) bilateral heterogeneous fatty areas in PGs (white arrows) on T1WI or CT; (**b**) bilateral heterogeneous fatty areas in SMGs (white arrows) on T1WI or CT; and (**c**) multiple bilateral hyperintense spots (<10 mm), suggesting punctate sialectasis in PGs (white arrows) on fsT2WI, short tau inversion recovery, or MR sialography. Arrows show the parotid glands (**a**,**c**) or the submandibular glands (**b**). T1WI, T1-weighted image; fsT2WI, fat-suppressed T2-weighted image; PGs, parotid glands; SMGs, submandibular glands.

The SS group comprised patients definitively diagnosed with SS by specialized rheumatologists based on the ACR/European League Against Rheumatism (EULAR) criteria [34,35]; patients who lacked items applicable to the ACR/EULAR criteria were definitively diagnosed according to the American–European Consensus Group (AECG), ACR, or the 1999 revised Japanese criteria for SS [36,37,38]. Of the patients who were not definitively diagnosed with SS because of a lack of necessary data, such as labial gland biopsy or autoantibody testing for SSA/Ro, for the abovementioned definitive diagnostic criteria, those who met the imaging criteria for SS (Figure 2) [6,7,8,9,10] were classified into the possible SS group. Whether each patient met the imaging criteria for SS was determined by consensus between two radiologists (Y.T. and M.SU.) with 26 years of experience in head and neck radiology. The patients who did not meet either the definitive diagnostic criteria or imaging criteria for SS were classified into the non-SS group.

### 2.4. Demographic and Imaging Findings

The age and sex of the patients were recorded. The following imaging findings were assessed for each lesion by consensus between the two radiologists (Y.T. and M.SU.): (i) the size (maximum diameter on axial images) and extent (simple/plunging ranulas) of the ranulas on MRI; (ii) the number and bilaterality of the parotid cysts on MRI; and (iii) the size (maximum diameter on axial bone algorithm images), number, bilaterality, and location (in Stensen’s duct/PG parenchyma including terminal ducts) [23,24] of the parotid calcifications on CT and the coexisting calcifications at other sites, such as the SMGs, vessels, pharyngeal walls, or lymph nodes.

### 2.5. Salivary Gland SS Disease Stage Score

**Figure 3:**
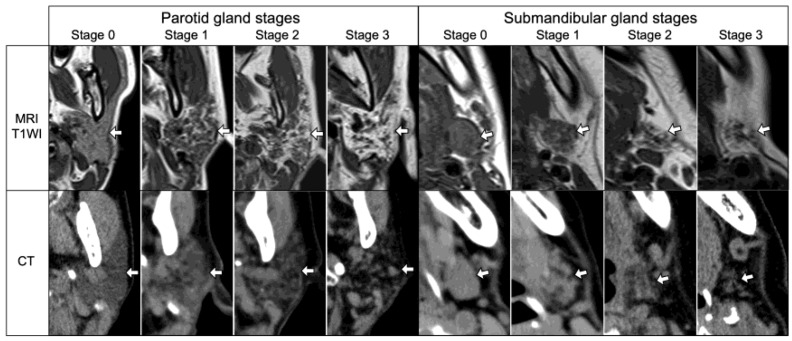
SS disease stage of parotid glands (white arrows) and submandibular glands (white arrows) on MRI and CT. Stage 0, no obvious fatty degeneration; Stage 1, weak fatty degeneration (up to 1/3 of the entire gland area); Stage 2, moderate fatty degeneration (more than 1/3 and up to 2/3 of the entire gland area); and Stage 3, severe fatty degeneration (over 2/3 of the entire gland area). T1WI, T1-weighted image.

Three radiologists (I.K., S.E., and M.SA.) with more than 24 years of experience in head and neck radiology who were blinded to the patient’s laboratory data examined the bilateral PGs and SMGs of each SS patient to qualitatively assess the salivary gland SS disease stage based on the fatty degeneration on the MRI (for patients with ranulas or parotid cysts) or CT (for patients with parotid calcifications). The disease stage score criteria were modified and simplified based on previously reported criteria [7,10] (Figure 3). If the disease stage of each gland differed bilaterally, a more severe stage was considered. Each patient’s PG and SMG stage scores were determined by calculating the average stage scores (0–3) assigned by the three radiologists.

### 2.6. Comparisons between the SS and Non-SS Groups and among Patients with the Three Lesion Types

The demographic and imaging findings of the SS and non-SS groups were compared. In addition, the age, PG, and SMG stage scores were compared among the patients with the three lesion types within the SS group, and age was compared among the patients with the three lesion types within the non-SS group.

### 2.7. Statistical Analysis

The prevalence of SS among the patients with ranulas, parotid cysts, or calcifications was compared using the chi-square test, followed by Bonferroni’s correction for multiple comparisons. To compare the variables of age, size, number of lesions, and SS stage score between the SS and non-SS groups, the Mann–Whitney U-test was used because of the non-normal distribution of these continuous variables (as determined by the Shapiro–Wilk normality test). Fisher’s exact test was used to compare other binary data. To assess the association with SS, we estimated the crude odds ratios (ORs) and 95% confidence intervals (CIs) using logistic regression analysis. The optimal cutoff values for the size or number of lesions to distinguish SS from non-SS were determined using receiver operating characteristic curve (ROC) analysis. The Steel–Dwass test was used for multiple pairwise comparisons of the age and salivary gland disease stage scores among the patients with the three lesion types. Interobserver agreement concerning the PG and SMG stage scores of the SS was assessed using Cohen’s weighted kappa coefficient. The agreement values for Cohen’s weighted kappa coefficient assessments were interpreted as follows: 0–0.2, poor agreement; >0.2 and ≤0.4, fair agreement; >0.4 and ≤0.6, moderate agreement; >0.6 and ≤0.8, substantial agreement; and >0.8 and ≤1.0, almost perfect agreement. Statistical significance was defined as *p* < 0.05. Statistical analyses were performed using the JMP Pro, version 16.2.0 (SAS Institute Inc., Cary, NC, USA), and IBM SPSS, version 27.0.1 (IBM, Armonk, NY, USA).

## 3. Results

Of the 228 patients with ranulas, parotid cysts, or parotid calcifications, 62 (27%), 40 (18%), and 126 (55%) were classified into the SS, possible SS, and non-SS groups, respectively (Figure 1 and Table 2). The SS group comprised the patients definitively diagnosed with SS based on the ACR/EULAR (*n* = 56), AECG (*n* = 58), ACR (*n* = 23), or 1999 revised Japanese criteria (*n* = 41). Of the 62 patients in the SS group, there were 35 and 27 primary SS and secondary SS patients, respectively (Supplementary Table S2), and 48 patients had already been diagnosed with SS before the imaging examinations (Table 2). Although the remaining 14 patients (7 with ranulas and 7 with parotid calcifications) were not suspected of having SS before the imaging examinations (Table 2), imaging findings characteristic of SS, such as fatty degeneration and/or punctate sialectasis of the salivary glands, were detected in addition to ranulas or parotid calcifications. Consequently, they were referred to rheumatologists for further examinations and were definitively diagnosed with pSS.

The prevalence rates of SS among the patients with ranulas, parotid cysts, or calcifications were 16%, 24%, and 40%, respectively. The prevalence of SS in the patients with parotid calcifications was significantly higher than that in the patients with ranulas (*p* = 0.0008) (Supplementary Table S3). In addition, the rates of either SS or possible SS were 25%, 41%, and 64% among the patients with ranulas, parotid cysts, or calcifications, respectively; among the female patients only, these rates were 33%, 46%, and 80%, respectively.

### 3.1. Prevalence of SS in Patients with Each Lesion Type and Comparison of the Demographic and Imaging Findings between the SS and Non-SS Groups

#### 3.1.1. Ranulas

**Figure 4:**
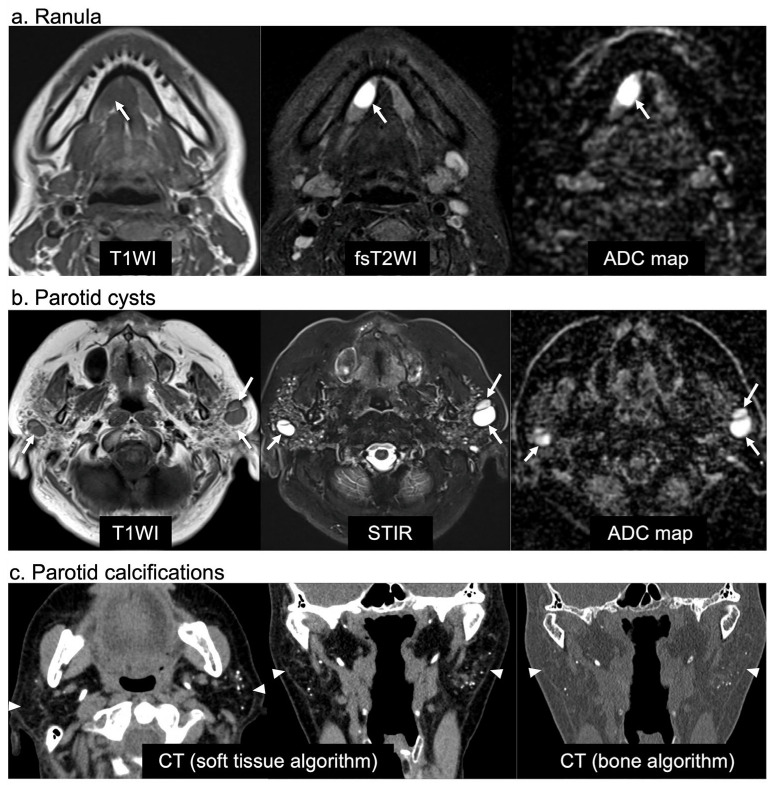
MR and CT images of SS patients. (**a**) Axial MR images of a 32-year-old woman with a right simple ranula (arrows). The ranula is a marked and homogeneous hyperintense area on fsT2WI with a high ADC (2.3 × 10^−3^ mm^2^/s). (**b**) Axial MR images of a 62-year-old woman with multiple bilateral parotid cysts (arrows) and a high ADC (2.0–2.1 × 10^−3^ mm^2^/s). The bilateral parotid glands also exhibited heterogeneous fatty areas on the T1WI and multiple hyperintense spots on the STIR image. (**c**) Axial and coronal CT images of a 68-year-old woman with multiple punctate calcifications in the bilateral parotid parenchyma (arrows). The bilateral parotid glands exhibit marked fatty degeneration. T1WI, T1-weighted image; fsT2WI, fat-suppressed T2-weighted image; ADC, apparent diffusion coefficient; STIR, short tau inversion recovery.

Of the 73 patients with ranulas on MRI, 12 (16%), 6 (8%), and 55 (75%) were classified into the SS, possible SS, and non-SS groups, respectively (Figure 1 and Table 2). A total of 7 (58%) of the 12 SS patients with ranulas were not suspected of having SS before the MRI examinations; ranula detection led to the detection of pSS (Table 2). In total, 11 (92%) of the 12 SS patients were females with simple ranulas, although there was no significant difference between the SS and non-SS groups regarding sex and a simple/plunging ranula (Table 3). The maximum ranula diameter was significantly smaller in the SS patients than in the non-SS patients (*p* = 0.045), and a small ranula (the optimal cutoff size from ROC analysis: maximum diameter ≤ 17 mm) was associated with SS (OR 5.25, 95% CI 1.27–21.66, *p* = 0.02) (Table 3 and Figure 4).

#### 3.1.2. Parotid Cysts

Of the 51 patients with parotid cysts on MRI, 12 (24%), 9 (18%), and 30 (59%) were classified into the SS, possible SS, and non-SS groups, respectively (Figure 1 and Table 2). There was no significant difference between the number of females in the SS group (92%) and that in the non-SS group (70%) (Table 3). Bilateral parotid cysts were present in 33% and 0% of the patients in the SS and non-SS groups, respectively (*p* < 0.001). In addition, the SS group had more multiple (*p* = 0.001) parotid cysts than the non-SS group. SS was significantly associated with multiple parotid cysts (OR 20.7, 95% CI 2.08–206.65, *p* = 0.01) and bilateral cysts (OR 32.29, 95% CI 1.58–661.1, *p* = 0.02) (Table 3 and Figure 4).

#### 3.1.3. Parotid Calcifications

Of the 116 patients with parotid calcifications on CT, 46 (40%), 28 (24%), and 42 (36%) were classified into the SS, possible SS, and non-SS groups, respectively (Figure 1 and Table 2). In total, 7 (15%) of the 46 SS patients with parotid calcifications were not suspected to have SS before CT examination; the detection of parotid calcifications led to the suspicion of SS (Table 2). The percentage of females was significantly higher in the SS group (98%) than in the non-SS group (43%) (*p* < 0.001) (Table 3). In addition, compared with the parotid calcifications in the non-SS group, those in the SS group were significantly more commonly bilateral (*p* < 0.001), multiple (*p* < 0.001), and located in the PG parenchyma (*p* = 0.03). Furthermore, the SS group had significantly fewer calcifications coexisting in other tissues than those in the non-SS group (*p* = 0.01). The parotid calcification findings significantly associated with SS were female sex (OR 60, 95% CI 7.54–477.26, *p* < 0.001), bilateral calcifications (OR 15.23, 95% CI 5.19–44.69, *p* < 0.001), calcifications located in the PG parenchyma (OR 8.99, 95% CI 1.06–76.6, *p* = 0.04), multiple calcifications (OR 7.43, 95% CI 2.70–20.40, *p* < 0.001), and a lack of coexisting calcifications in other tissues (OR 0.31, 95% CI 0.12–0.77, *p* = 0.01) (Table 3 and Figure 4).

### 3.2. Comparisons between Patients with the Three Lesion Types

**Figure 5:**
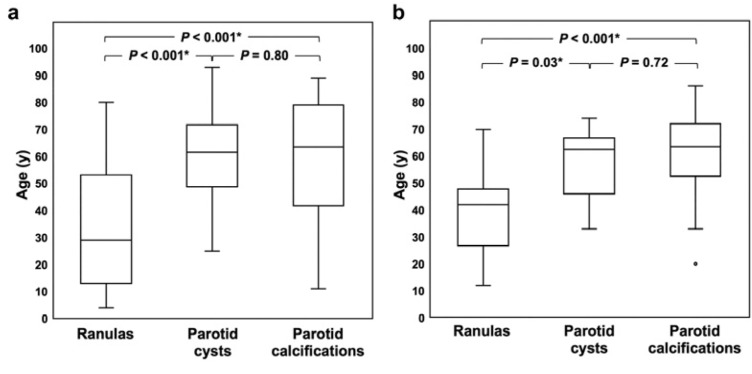

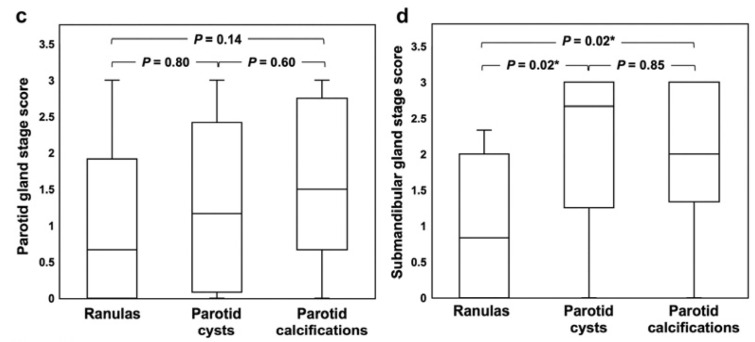
Comparisons between patients with the three lesion types. (**a**) Comparison of the ages between non-SS patients with the three lesion types; (**b**) comparison of the ages between SS patients with the three lesion types; (**c**) comparison of parotid gland stage scores between SS patients with the three lesion types; (**d**) comparison of submandibular gland stage scores between SS patients with the three lesion types. The horizontal lines represent the median (50th percentile) values; the top and bottom of the boxes represent the 25th and 75th percentiles, respectively; the whiskers indicate the range from the largest and smallest observed data within the 1.5 interquartile range presented by a box. *p*-values obtained by the Steel–Dwass test. * Statistically significant results.

The patients with ranulas were significantly younger than those with parotid cysts or parotid calcifications in the non-SS (*p* < 0.001 for both) (Figure 5a) and SS groups (*p* = 0.03 and *p* < 0.001, respectively) (Figure 5b). The median (interquartile range) PG/SMG stage scores of the SS patients with ranulas, parotid cysts, or parotid calcifications were 0.67 (0–1.92)/0.83 (0–2.0), 1.17 (0.08–2.42)/2.70 (1.25–3), and 1.50 (0.67–2.75)/2.00 (1.33–3), respectively. The patients with ranulas had significantly lower SMG stage scores than those with parotid cysts or parotid calcifications (*p* = 0.02 for both) (Figure 5d). The patients with ranulas tended to have lower PG stage scores than those with parotid calcifications, although the difference was not statistically significant (*p* = 0.14) (Figure 5c). There was no difference between the patients with parotid cysts and those with parotid calcifications in the PG or SMG stage scores (*p* = 0.60 and *p* = 0.85, respectively) (Figure 5c,d).

### 3.3. Interobserver Agreements of PG and SMG Stage Scores

Cohen’s weighted kappa coefficients of the interobserver agreements regarding the PG and SMG stage scores indicated substantial to almost perfect agreement between the three radiologists (0.72–0.84) (Supplementary Table S4).

## 4. Discussion

To the best of our knowledge, this is the first study to retrospectively investigate the prevalence of SS among patients with ranulas, parotid cysts, or parotid calcifications; compare the MRI or CT findings of each lesion between SS and non-SS patients; and compare the age and salivary gland disease stages among SS patients with the three lesion types. This study found that the prevalence of SS among the patients with ranulas, parotid cysts, or parotid calcifications was 16%, 24%, and 40%, respectively, with a particularly high prevalence in the patients with parotid calcifications. Some possible SS patients may be definitively diagnosed with SS in the future through required examinations, such as labial gland biopsy or autoantibody testing for SSA/Ro; therefore, the true frequencies of SS among patients with the lesions are expected to be much higher. Assuming that all possible SS patients were definitively diagnosed with SS after the necessary examinations, our results indicated that 25%, 41%, and 64% of the patients with ranulas, parotid cysts, and calcifications (33%, 46%, and 80% of the female patients with ranulas, parotid cysts, and calcifications, respectively) had SS. Furthermore, the findings significantly associated with SS were as follows, (i) ranula: ≤17 mm; (ii) parotid cysts: bilateral and multiple; and (iii) parotid calcifications: in females, bilateral, multiple, parenchymal, and no coexisting calcifications in other tissues.

The presence of bilateral or multiple findings in both parotid cysts and parotid calcifications was found to be highly suggestive of SS, which is consistent with previous studies [16,17,18,19,20,22,23,24,25]. Additionally, parotid calcifications in the PG parenchyma or those without coexisting calcifications in other tissues were significantly associated with SS. This suggests that parotid calcifications in SS are associated with chronic inflammation from autoantibody-driven lymphocyte infiltration and represent a separate condition from intraductal obstructive sialolithiasis and dystrophic calcifications [24,39].

Ranulas occur frequently in younger patients [28,29]. In the SS and non-SS groups in the present study, the patients with ranulas were significantly younger than those with parotid cysts or parotid calcifications. However, the ranulas in the SS patients were significantly smaller than those in the non-SS patients, possibly because of the lower salivary flow. Furthermore, the median PG and SMG stage scores of the SS patients with ranulas were <1, and the SMG scores were significantly lower than those of the SS patients with parotid cysts or parotid calcifications, suggesting that ranulas are more common than parotid cysts or calcifications in early-stage SS. This supports the hypothesis that ranulas occur in the early stages of SS because acinar atrophy, loss, and rupture due to lymphocyte aggregation in advanced SS further decrease the salivary flow, resulting in insufficient saliva to form a ranula [28]. The prevalence of SS in the patients with ranulas (16%) was significantly lower than that in the patients with parotid calcifications (40%). However, the rate of SS or possible SS among the female patients with ranulas was 33%, which was not negligible. Additionally, in 58% of the SS patients with ranulas, the detection of the ranulas led to the diagnosis of pSS at a relatively young age (average age 35 years). Therefore, recognizing that one-third of females with ranulas may have SS could aid in detecting pSS at younger or earlier stages.

Our findings indicate that small ranulas, bilateral or multiple parotid cysts, and bilateral or multiple parotid calcifications are important signs of SS. However, the same findings have also been reported in cases of human immunodeficiency virus (HIV) infection [24,40,41,42,43,44]. HIV infection is associated with xerostomia related to hypofunction of the salivary glands and therefore has highly similar clinical and imaging findings to SS [43], although HIV infection is more common in males [43]. In addition, Syebele et al. reported that 48.6% (35/72) of ranulas in patients with HIV were plunging [44], whereas in the SS patients with ranulas in our study, only 8% (1/12) were plunging ranulas. Furthermore, an immunobiological study of cystic benign lymphoepithelial lesions of the PGs reported that CD4+ helper T cells/CD8+ suppressor T cells were markedly decreased in patients with HIV, whereas CD4/CD8 concentrations were normal in SS [26]. In the present study, none of the SS patients had an HIV infection. However, because the patients in the non-SS and possible SS groups did not undergo HIV serological testing, some may have been infected with HIV.

This study had some limitations. First, because this was a retrospective study, (i) it was not possible to apply definitive diagnostic criteria for SS to all the subjects, (ii) four different definitive diagnostic criteria (ACR/EULAR, AECG, ACR, and the 1999 revised Japanese criteria) were utilized, and (iii) imaging criteria were employed for patients who lacked the necessary items for a definitive diagnosis; however, 3 of the 62 SS patients did not meet the imaging criteria for SS, indicating that the sensitivity of the imaging criteria was 95% and not completely accurate for the diagnosis of SS (Table 2). Therefore, not only in the possible SS group but also some patients in the non-SS group may have been diagnosed with SS when the definitive diagnostic criteria have been applied. The number of SS in this study was likely underestimated. In the future, a prospective study should be conducted for patients with ranulas, parotid cysts, or calcifications found on MRI and/or CT to definitively diagnose SS or non-SS using the 2016 ACR/EULAR classification criteria, the latest criteria for SS. Second, because this was a single-center study, the number of patients was small, especially in the SS group, which prevented sufficient statistical analysis, including multivariate analysis. A multicenter prospective study is required to clarify the exact prevalence of SS and the imaging findings associated with SS, which may allow us to propose new reliable diagnostic criteria for SS using ranulas, parotid cysts, or calcifications. Third, most parotid cysts and calcifications in this study were followed up without a definitive histopathological diagnosis. This is because most of these cases were asymptomatic and were discovered incidentally on imaging examinations, and there is a risk of facial nerve paralysis when performing parotid biopsy or excision. Therefore, the parotid cysts detected in this study may have been other cystic conditions associated with parotid tumors, such as cystic Warthin tumors, pleomorphic adenomas, low-grade mucoepidermoid carcinomas, and adenoid cystic carcinomas [20,33]. Fourth, although lymphoepithelial lesions associated with SS include complete cystic lesions as well as many combined cystic and solid lesions or complete solid lesions [21,45,46], only cystic lesions detected on MRI were included because it is difficult to differentiate between lymphoepithelial lesions, other neoplastic lesions, or inflammatory lesions without a histopathologic diagnosis. However, the aggregation and proliferation of benign lymphoepithelial lesions can lead to acquired mucosa-associated lymphoid tissue lymphoma [26], and lymphoepithelial lesions were recently reported to be present in half of the patients with pSS [47]. Therefore, all parotid gland mass lesions, and not only complete cystic lesions, should be investigated in the future.

## 5. Conclusions

Our study revealed that the prevalence of SS among the patients with ranulas, parotid cysts, or parotid calcifications was 16%, 24%, and 40%, respectively. Additionally, including the SS patients and possible SS patients, 25%, 41%, and 64% of the patients with ranulas, parotid cysts, or calcifications (33%, 46%, and 80% of the female patients with ranulas, parotid cysts, or calcifications), respectively, were suspected of having SS. Furthermore, the following were significantly associated with SS: (i) ranula: ≤17 mm; (ii) parotid cysts: bilateral and multiple; and (iii) parotid calcifications: in females, bilateral, multiple, parenchymal, and no coexisting calcifications in other tissues.

Currently, the most well-recognized characteristic MRI and CT imaging findings of SS are heterogeneous fatty degeneration in the PGs and/or SMGs on MRI and/or CT and punctate sialectasis in PGs on MRI [6,7,8,9,10]. However, punctate sialectasis can only be detected on MR sialography, fsT2WI, or STIR, and the fatty degeneration of salivary glands found incidentally is often not noted because it is also observed in older adults and in patients with hyperlipidemia [9,48,49,50]. Furthermore, these findings are often overlooked when the changes are slight. In contrast, ranulas, parotid cysts, and parotid calcifications on CT or MRI are more noticeable and easily detected. Additionally, in 58% and 15% of the SS patients with ranulas and parotid calcifications, respectively, detection of the lesions led to the diagnosis of primary SS. Therefore, recognition of the prevalence of SS in patients with the lesions and characteristic imaging findings of the lesions associated with SS may promote referral to rheumatologists and facilitate the detection of undiagnosed pSS. In particular, a ranula is suggested to be an early stage sign of SS; thus, this might aid in the early detection of pSS in younger patients.

## Figures and Tables

**Table 1:** Inclusion and exclusion criteria for ranulas, parotid cysts, and parotid calcifications.

**Ranulas**				
	Inclusion criteria			
		Confirmed diagnosis based on histopathology		
		Clinical diagnosis based on both clinical and MRI findings ^§^		
			MRI criteria (meeting a–c)	
				(a) Well-defined cystic areas within or connected to sublingual glands
				(b) Marked and homogeneous hyperintense areas on fsT2WI or STIR
				(c) High ADC areas (≥2.0 × 10^−3^ mm^2^/s) [31,32]
	Exclusion criteria			
		No evaluable MR images ^†^		
		Difficulty in differentiating from other lesions ^(i)^		
**Parotid cysts**				
	Inclusion criteria			
		Confirmed diagnosis based on histopathology		
		Clinical diagnosis based on both clinical and MRI findings ^§^		
			MRI criteria (meeting a–d)	
				(a) Well-defined cystic areas with no solid portion in PGs
				(b) Maximum diameter ≥ 10 mm
				(c) Marked and homogeneous hyperintense areas on fsT2WI or STIR
				(d) High ADC areas (≥2.0 × 10^−3^ mm^2^/s) [31]
	Exclusion criteria			
		No evaluable MR images ^†^		
		Difficulty in differentiating from other lesions ^(ii)^		
**Parotid calcifications**				
	Inclusion criteria			
		Confirmed diagnosis based on histopathology		
		Clinical diagnosis based on both clinical and CT findings ^§^		
			CT criteria	
				Calcifications (>100 H.U.) in PGs on non-contrast enhanced CT
	Exclusion criteria			
		Calcifications associated with mass lesions ^(iii)^		
		Calcifications in patients who previously underwent X-ray parotid gland sialography ^‡^		

^§^ Not confirmed histopathologically but clinically diagnosed and regularly followed up. ^†^ Due to a lack of MRI examinations or severe artifacts. ^(i)^ Lymphangiomas, hemangiomas, or dermoid cysts. ^(ii)^ Cystic Warthin tumors, pleomorphic adenomas, low-grade mucoepidermoid carcinomas, other tumors, or lymph node disease [20,33]. ^(iii)^ Tumors and vascular malformations. ^‡^ Patients with impaired salivary excretory function (as in SS) may retain contrast medium showing a high density in the parotid gland long after the examination [23]. fsT2WI, fat-suppressed T2-weighted imaging; STIR, short tau inversion recovery; ADC, apparent diffusion coefficient; PGs, parotid glands.

**Table 2:** Demographic, clinical, and imaging findings of SS, possible SS, and non-SS groups.

	SS group 62 patients 70 lesions	Possible SS group 40 patients 43 lesions	Non-SS group 126 patients 127 lesions
**Ranulas on MRI (73 patients)**			
Number of patients, *n* (%)	12 (16)	6 (8)	55 (75)
Median age (IQR), y	42 (26.8–47.8)	30 (24.3–44.8)	29 (13–53)
Female, *n* (%)	11 (92)	6 (100)	34 (62)
Histopathologically confirmed, *n* (%)	3 (25)	2 (33)	21 (38)
Prior to diagnosis of SS ^†^, *n* (%)	7 (58)	-	-
Primary SS/secondary SS	10/2	-	-
Imaging criteria for SS			
(a) Bilateral heterogeneous fat deposition in PGs on T1WI, *n* (%)	7 (58)	2 (33)	0
(b) Bilateral heterogeneous fat deposition in SMGs on T1WI, *n* (%)	7 (58)	2 (33)	0
(c) Bilateral multiple hyperintense spots in PGs on fsT2WI or STIR, *n* (%)	11 (92)	6 (100)	0
(d) Bilateral multiple hyperintense spots in PGs on MR sialography, *n* (%)	9 (9/9 = 100) *	6 (6/6 = 100) *	0 (0/6 = 0) *
Met any of (a), (b), (c), or (d) ^§^, *n* (%)	12 (100)	6 (100)	0
**Parotid cysts on MRI (51 patients)**			
Number of patients, *n* (%)	12 (24)	9 (18)	30 (59)
Median age (IQR), y	62.5 (46–66.8)	70 (54–80.5)	61.5 (48.8–71.8)
Female, *n* (%)	11 (92)	7 (78)	21 (70)
Histopathologically confirmed, *n* (%)	0	2 (22)	9 (33)
Prior to diagnosis of SS ^†^, *n* (%)	0	-	-
Primary SS/secondary SS	5/7	-	-
Imaging criteria for SS			
(a) Bilateral heterogeneous fat deposition in PGs on T1WI, *n* (%)	8 (67)	8 (89)	0
(b) Bilateral heterogeneous fat deposition in SMGs on T1WI, *n* (%)	11 (92)	8 (89)	0
(c) Bilateral multiple hyperintense spots in PGs on fsT2WI or STIR, *n* (%)	6 (50)	4 (44)	0
(d) Bilateral multiple hyperintense spots in PGs on MR sialography, *n* (%)	5 (5/6 = 83) *	3 (3/4 = 75) *	0 (0/8 = 0) *
Met any of (a), (b), (c), or (d) ^§^, *n* (%)	11 (92) ***	9 (100)	0
**Parotid calcifications on CT (116 patients)**			
Number of patients, *n* (%)	46 (40)	28 (24)	42 (36)
Median age (IQR), y	63.5 (52.5–72)	70 (62.5–78.8)	63.5 (41.8–79)
Female, *n* (%)	45 (98)	26 (93)	18 (43)
Histopathologically confirmed, *n* (%)	0	0	2 (5)
Prior to diagnosis of SS ^†^, *n* (%)	7 (15)	-	-
Primary SS/secondary SS	26/20	-	-
Imaging criteria for SS			
(a) Bilateral heterogeneous fat deposition in PGs on CT, *n* (%)	38 (83)	25 (89)	0
(b) Bilateral heterogeneous fat deposition in SMGs on CT, *n* (%)	38 (83)	25 (89)	0
(c) Bilateral multiple hyperintense spots in PGs on fsT2WI or STIR, *n* (%)	5 (5/8 = 63) **	1 (1/3 = 33) **	0 (0/1 = 0) **
(d) Bilateral multiple hyperintense spots in PGs on MR sialography, *n* (%)	5 (5/7 = 71) *	Not scanned	0 (0/1 = 0) *
Met any of (a), (b), (c), or (d) ^§^, *n* (%)	44 (96) ***	28 (100)	0

Numbers in the table indicate the number of patients with each finding (percentage of patients with each finding). * MR sialography was performed in only some patients. ** Only 12 patients out of all patients with parotid calcifications had evaluable MR images. *** A total of 1 patient with parotid cysts and 2 patients with parotid calcifications among 62 SS patients did not meet the imaging criteria for SS, indicating that the sensitivity of the imaging criteria for SS was 95%. ^†^ Patients who were not suspected of having SS before the imaging examinations. ^§^ Patients who did not meet the definitive diagnostic criteria for SS but met any of (a), (b), (c), or (d) were classified into the possible SS group. PGs, parotid glands; SMGs, submandibular glands; IQR, interquartile range; SD, standard deviation; T1WI, T1-weighted imaging; fsT2WI, fat-suppressed T2-weighted imaging; STIR, short tau inversion recovery.

**Table 3:** Comparison of demographic and imaging findings between the SS and non-SS groups.

		SS group 62 patients 70 lesions	Non-SS group 126 patients 127 lesions	Comparison between SS and non-SS groups *p-*value ^†^	Univariate logistic regression analysis OR (95% CI)/ *p*-value ^‡^
Ranulas on MRI (67 patients)					
Number of patients		12	55		
Median age (IQR), y		42 (26.8–47.8)	29 (13–53)	0.41	1.01 (0.98–1.04)/ 0.54
Female, *n* (%)		11 (92)	34 (62)	0.09	6.79 (0.82–56.50)/ 0.08
Imaging findings of ranulas					
	Median maximum diameter (IQR), mm	10 (6.3–26.8)	22 (10–31)	**0.045**	0.96 (0.91–1.01)/ 0.09
	(Maximum diameter ≤ 17 mm *, *n* (%))	9 (75)	20 (36)	**0.02**	**5.25 (1.27–21.66)/** **0.02**
	Simple ranulas/plunging ranulas (Percentage of simple ranulas)	11/1 (92)	44/11 (80)	0.68	2.75 (0.32–23.63)/ 0.36
Parotid cysts on MRI (42 patients)					
Number of patients		12	30		
Median age (IQR), y		62.5 (46–66.8)	61.5 (48.8–71.8)	0.69	0.67 (0.03–12.94)/ 0.79
Female, *n* (%)		11 (92)	21 (70)	0.23	4.71 (0.53–42.16)/ 0.17
Imaging findings of parotid cysts					
	Median number of cysts (IQR)	1 (1–3)	1 (1–1)	**0.001**	**8.53 (1.18–61.76)/** **<0.001**
	(Number of cysts ≥ 2 *, *n* (%))	5 (42)	1 (3)	**0.005**	**20.7 (2.08** **–** **206.65)/** **0.01**
	Bilaterality, *n* (%)	4 (33)	0 (0)	**<0.001**	**32.29 (1.58–661.1)/** **0.02** **^§^**
Parotid calcifications on CT (88 patients)					
Number of patients		46	42		
Median age (IQR), y		63.5 (52.5–72)	63.5 (41.8–79)	0.85	1.01 (0.99–1.03)/ 0.37
Female, *n* (%)		45 (98)	18 (43)	**<0.001**	**60 (7.54–477.26)/** **<0.001**
Imaging findings of parotid calcifications					
	Median number of calcifications (IQR)	5.5 (2–15)	1 (1–3)	**<0.001**	**1.22 (1.07–1.39)/** **<0.001**
	(Number of calcifications ≥ 2 *, *n* (%))	39 (85)	18 (43)	**<0.001**	**7.43 (2.70–20.40)/** **<0.001**
	Bilaterality, *n* (%)	33 (72)	6 (14)	**<0.001**	**15.23 (5.19** **–** **44.69)/** **<0.001**
	Punctate calcification (<2 mm), *n* (%)	31 (67)	25 (60)	0.51	1.41 (0.59–3.36)/ 0.44
	PG parenchymal calcification, *n* (%)	45 (98)	35 (83)	**0.03**	**8.99 (1.06–76.6)/** **0.04**
	Coexistence calcification in SMGs, *n* (%)	6 (13)	7 (17)	0.77	0.75 (0.23–2.44)/ 0.63
	Coexisting calcification in other tissues, *n* (%)	10 (22)	20 (48)	**0.01**	**0.31 (0.12–0.77)/** **0.01**

Numbers in bold indicate statistically significant results. ^†^ Mann–Whitney U-test for age, size, and number of lesions or Fisher’s exact test for other variables. ^‡^ (SS group/non-SS group) with each finding/(SS group/non-SS group) without each finding. * The optimal cutoff value was determined using receiver operating characteristic curve analysis. ^§^ Odds ratios (ORs) of contingency tables with 0 counts were corrected using the Haldane–Anscombe method. PG, parotid gland; SMGs, submandibular glands; OR, odds ratio; CI, confidence interval; IQR, interquartile range.

## Data Availability

The data that support the findings of this study are available from the corresponding author, M.SU., upon reasonable request.

## Supplementary Materials

The following supporting information can be downloaded at https://www.mdpi.com/article/10.3390/jcm12206487/s1, Supplementary Table S1: Magnetic resonance imaging parameters; Supplementary Table S2: Demographic, clinical, and imaging findings of primary and secondary SS; Supplementary Table S3: Comparison of the prevalence of SS among patients with ranulas, parotid cysts, and parotid calcifications; Supplementary Table S4: Interobserver agreement concerning the PG and SMG stage scores of SS patients.
