# Supplementary material for: Three Signs to Help Detect Sjögren’s Syndrome: Incidental Findings on Magnetic Resonance Imaging and Computed Tomography
Source: J Clin Med. 2023 Oct 12;12(20):6487. doi: 10.3390/jcm12206487 (PMC10607120; doi:10.3390/jcm12206487)
Supplement: Supplementary file 1 [file jcm-12-06487-s001.zip › jcm-2617616-supplementary.pdf]

Supplementary Table S1. Magnetic resonance imaging parameters.

T1-weighted imaging (T1WI)	Fat-suppressed T2-weighted imaging (fsT2WI)	Short tau inversion recovery (STIR)	Diffusion-weighted imaging	MR sialography
**1.5 T MRI**				
TR/TE/ TSE factor /NEX = 500 ms/15 ms/3/2<br>FOV/acquisition matrix/thickness/gap = 200 mm/256 × 204 /4 mm/0.4 mm	TR/TE/TSE factor/NEX = 6385 ms/80 ms/15/2<br>FOV/acquisition matrix/ thickness/gap = 200 mm/256 × 204/4mm/ 0.4 mm<br>Fat-suppression using SPAIR	TR/TE/TI/NEX = 4000 ms/80 ms/180 ms/2<br>FOV/acquisition matrix/ thickness/gap = 200 mm/192 × 143/ 4 mm/0.4 mm	Single-shot EPI<br>TR/TE/NEX = 4283ms/87 ms/4<br>FOV/acquisition matrix/ thickness gap = 200 mm/112 × 90/ 4 mm/0.4 mm<br>b value = 0, 500, 1000 s/mm²	TR/TE/ TSE factor/NEX = 8000 ms/800 ms/54/4<br>FOV/acquisition matrix/thickness = 80 mm/192 × 189/ 35 mm<br>Fat-suppression using SPAIR
**3.0 T MRI**				
**T1-weighted imaging (T1WI)**	**Fat-suppressed T2-weighted imaging (fsT2WI)**	**Short tau inversion recovery (STIR)**	**Diffusion-weighted imaging**	**MR sialography**
TR/TE/TSE factor/ NEX = 420 ms/9 ms/3/2<br>FOV/acquisition matrix/thickness/gap = 200 mm/320 × 208/ 4 mm/0.4 mm	TR/TE/TSE factor/NEX = 3800 ms/86 ms/12/2<br>FOV/acquisition matrix/ thickness/gap = 200 mm/384 × 230/ 4 mm/0.4 mm<br>Fat-suppression using Dixon	TR/TE/TI/NEX = 5520 ms/67 ms/230 ms/2<br>FOV/acquisition matrix/thickness/gap = 200 mm/320 × 224 /4 mm/0.4 mm	Readout-segmented EPI<br>TR/TE/NEX = 3800 ms/86 ms/1<br>FOV/acquisition matrix/ thickness/gap = 220 mm/160 × 160/ 5 mm/0.5 mm<br>b value = 0, 500, 1000 s/mm²	TR/TE/TSE factor/NEX = 8000 ms/1100 ms/361/6<br>FOV/acquisition matrix/thickness = 123 mm/ 320 × 224/35 mm<br>Fat-suppression using SPAIR

TR, repetition time; TE, echo time; TSE: turbo spin echo; NEX: number of excitations; FOV: field of view; TI, inversion time; SPAIR, spectral attenuation with inversion recovery; EPI, echo-planar imaging.

Supplementary Table S2. Demographic, clinical, and imaging findings of primary and secondary SS.

	**Primary SS**<br>35 patients<br>41 lesions	**Secondary SS**<br>27 patients<br>29 lesions
[redacted]		
Number of patients	10	2
Median age (IQR) y	44.5 (30.3–48.8)	26.5 (12–41)
Female, n (%)	9 (90)	2 (100)
Histopathologically confirmed, n (%)	2 (20)	1 (50)
Prior to diagnosis of SS†, n (%)	7 (70)	0
Imaging criteria for SS		
(a) Bilateral heterogeneous fat deposition in PGs on T1WI, n (%)	6 (60)	1 (50)
(b) Bilateral heterogeneous fat deposition in SMGs on T1WI, n (%)	6 (60)	1 (50)
(c) Bilateral multiple hyperintense spots in PGs on fsT2WI or STIR, n (%)	9 (90)	2 (100)
(d) Bilateral multiple hyperintense spots in PGs on MR sialography, n (%)	9 (9/9 = 100)	0
Met any of (a), (b), (c), or (d) §, n (%)	10 (100)	2 (100)
Anti- Ro/SSA antibody, n (%)	8 (80)	2 (100)
Anti-La/SSB antibody, n (%)	2 (2/9 = 22)	1 (50)
Antinucler antibody, n (%)	8 (8/8 = 100)	2 (100)
Rheumatoid factor, n (%)	7 (7/8 = 88)	2 (100)
[redacted]		
Number of patients	5	7
Median age (IQR) y	63 (39–70.5)	62 (49–66)
Female, n (%)	5 (100)	6 (86)
Histopathologically confirmed, n (%)	0	0
Prior to diagnosis of SS†, n (%)	0	0
Imaging criteria for SS		
(a) Bilateral heterogeneous fat deposition in PGs on T1WI, n (%)	4 (80)	4 (57)
(b) Bilateral heterogeneous fat deposition in SMGs on T1WI, n (%)	5 (100)	6 (86)
(c) Bilateral multiple hyperintense spots in PGs on fsT2WI or STIR, n (%)	3 (60)	3 (43)
(d) Bilateral multiple hyperintense spots in PGs on MR sialography, n (%)	2 (2/3 = 67)	3 (3/3 = 100)
Met any of (a), (b), (c), or (d) §, n (%)	5 (100)	6 (86)
Anti- Ro/SSA antibody, n (%)	3 (3/5 = 60)	5 (5/6 = 83)
Anti-La/SSB antibody, n (%)	0	3 (3/6 = 50)
Antinucler antibody, n (%)	3 (3/5 = 60)	4 (4/6 = 67)
Rheumatoid factor, n (%)	1 (1/2 = 50)	4 (4/4 = 100)
[redacted]		
Number of patients	26	20
Median age (IQR) y	66 (57.8–69)	59 (49–72.8)
Female, n (%)	26 (100)	19 (95)
Histopathologically confirmed, n (%)	0	0
Prior to diagnosis of SS†, n (%)	6 (23)	1 (5)

Imaging criteria for SS		
(a) Bilateral heterogeneous fat deposition in PGs on CT, n (%)	23 (88)	15 (75)
(b) Bilateral heterogeneous fat deposition in SMGs on CT, n (%)	22 (85)	16 (80)
(c) Bilateral multiple hyperintense spots in PGs on fsT2WI or STIR, n (%)	4 (4/6 = 67)	1 (1/2 = 50)
(d) Bilateral multiple hyperintense spots in PGs on MR sialography, n (%)	4 (4/5 = 80)	1 (1/2 = 50)
Met any of (a), (b), (c), or (d) §, n (%)	25 (96)	19 (95)
Anti- Ro/SSA antibody, n (%)	19 (19/24 = 79)	17 (17/18 = 94)
Anti-La/SSB antibody, n (%)	6 (6/23 = 26)	8 (8/17 = 47)
Antinucler antibody, n (%)	15 (15/21 = 71)	11 (11/13 = 85)
Rheumatoid factor, n (%)	13 (13/16 = 81)	11 (11/14 = 79)

Numbers in the table indicate the number of patients with each finding (percentage of patients with each finding).

†Patients who were not suspected of having SS before the imaging examinations.

§Patients who did not meet the definitive diagnostic criteria for SS but met any of (a), (b), (c), or (d) were classified into the possible SS group.

PGs, parotid glands; SMGs, submandibular glands; IQR, interquartile range; SD, standard deviation; T1WI, T1-weighted imaging; fsT2WI, fat-suppressed T2-weighted imaging; STIR, short tau inversion recovery.

Supplementary Table S3. Comparison of the prevalence of SS among patients with ranulas, parotid cysts, or parotid calcifications.

	P-value
Ranulas vs Parotid cysts	0.3254
Ranulas vs Parotid calcifications	**0.0008**
Parotid cysts vs Parotid calcifications	0.0438

Numbers indicate *p*-values by the chi-square test. According to Bonferroni's correction for multiple comparisons, a $p < 0.01667$ (=0.05/3) was considered statistically significant. Numbers in bold indicate statistically significant results.

Supplementary Table S4. Interobserver agreement concerning the PG and SMG stage scores of SS patients.

Observers	PG stage score	SMG stage score
Radiologist A vs B	0.77	0.72
Radiologist A vs C	0.84	0.73
Radiologist B vs C	0.82	0.79

Numbers indicate Cohen's weighted kappa coefficients.

PG, parotid gland; SMG, submandibular gland.

Agreement values for Cohen's weighted kappa coefficient assessments were interpreted as follows: 0–0.2, poor agreement; > 0.2 and ≤ 0.4, fair agreement; > 0.4 and ≤ 0.6, moderate agreement; > 0.6 and ≤ 0.8, substantial agreement; and > 0.8 and ≤ 1.0, almost perfect agreement.
